# Causes and circumstances of maternal death: a secondary analysis of the Community-Level Interventions for Pre-eclampsia (CLIP) trials cohort

**DOI:** 10.1016/S2214-109X(21)00263-1

**Published:** 2021-07-29

**Authors:** Annet M Aukes, Kristina Arion, Jeffrey N Bone, Jing Li, Marianne Vidler, Mrutyunjaya B Bellad, Umesh Charantimath, Shivaprasad S Goudar, Zahra Hoodbhoy, Geetanjali Katageri, Salésio Macuacua, Ashalata A Mallapur, Khátia Munguambe, Rahat N Qureshi, Charfudin Sacoor, Esperança Sevene, Sana Sheikh, Anifa Valá, Gwyneth Lewis, Zulfiqar A Bhutta, Peter von Dadelszen, Laura A Magee, CLIP Trials Study Group, Mai-Lei Woo Kinshella, Hubert Wong, Peter von Dadelszen, Faustino Vilanculo, Marianne Vidler, Anifa Valá, Ugochi V Ukah, Domena K Tu, Lehana Thabane, Corsino Tchavana, Jim Thornton, John O Sotunsa, Joel Singer, Sana Sheikh, Sumedha Sharma, Esperança Sevene, Nadine Schuurman, Diane Sawchuck, Charfudin Sacoor, Amit P Revankar, Farrukh Raza, Umesh Y Ramdurg, Rahat N Qureshi, Rosa Pires, Beth A Payne, Vivalde Nobela, Cláudio Nkumbula, Ariel Nhancolo, Zefanias Nhamirre, Khátia Munguambe, Geetanjali I Mungarwadi, Dulce Mulungo, Sibone Mocumbi, Craig Mitton, Mario Merialdi, Javed Memon, Analisa Matavele, Sphoorthi S Mastiholi, Ernesto Mandlate, Ashalata A Mallapur, Laura A Magee, Sónia Maculuve, Salésio Macuacua, Eusébio Macete, Marta Macamo, Mansun Lui, Jing Li, Gwyneth Lewis, Simon Lewin, Tang Lee, Ana Langer, Uday S Kudachi, Bhalachandra S Kodkany, Marian Knight, Gudadayya S Kengapur, Avinash J Kavi, Geetanjali Katageri, Chirag Kariya, Chandrappa C Karadiguddi, Namdev A Kamble, Anjali M Joshi, Eileen Hutton, Amjad Hussain, Narayan V Honnungar, Zahra Hoodbhoy, William Grobman, Shivaprasad S Goudar, Emília Gonçálves, Tabassum Firoz, Veronique Fillipi, Paulo Filimone, Susheela M Engelbrecht, Dustin T Dunsmuir, Guy Dumont, Sharla K Drebit, France Donnay, Shafik Dharamsi, Vaibhav B Dhamanekar, Richard Derman, Brian Darlow, Silvestre Cutana, Keval S Chougala, Rogério Chiaú, Umesh Charantimath, Romano Nkumbwa Byaruhanga, Jeffrey N Bone, Helena Boene, Ana Ilda Biz, Cassimo Bique, Zulfiqar A Bhutta, Ana Pilar Betrán, Mrutyunjaya B Bellad, Shashidhar G Bannale, Annet M Aukes, Orvalho Augusto, Kristina Arion, J Mark Ansermino, Felizarda Amose, Imran Ahmed, Olalekan O Adetoro

**Affiliations:** a Department of Obstetrics and Gynaecology, BC Children's Hospital Research Institute, University of British Columbia, Vancouver, BC, Canada; b Department of Obstetrics and Gynaecology, University Medical Center Groningen, Groningen, Netherlands; c Department of Obstetrics and Gynaecology, IWK Health Centre, Dalhousie University, Halifax, NS Canada; d KLE Academy of Higher Education and Research, Jawaharlal Nehru Medical College, Belagavi, Karnataka, India; e Centre of Excellence, Division of Woman and Child Health, Aga Khan University, Karachi, Pakistan; f S Nijalingappa Medical College, Hanagal Shree Kumareshwar Hospital and Research Centre, Bagalkot, Karnataka, India; g Centro de Investigação em Saúde da Manhiça, Manhiça, Mozambique; h Department of Physiological Sciences, Clinical Pharmacology, Faculdade de Medicina, Universidade Eduardo Mondlane, Maputo, Mozambique; i Faculty of Health and Social Sciences, Bournemouth University, Bournemouth, UK; j Centre for Global Child Health, Hospital for Sick Children, Toronto, ON, Canada; k Department of Women and Children's Health, School of Life Course Sciences, Faculty of Life Sciences and Medicine, King's College London, London, UK

## Abstract

**Background:**

Incomplete vital registration systems mean that causes of death during pregnancy and childbirth are poorly understood in low-income and middle-income countries. To inform global efforts to reduce maternal mortality, we compared physician review and computerised analysis of verbal autopsies (interpreting verbal autopsies [InterVA] software), to understand their agreement on maternal cause of death and circumstances of mortality categories (COMCATs) in the Community-Level Interventions for Pre-eclampsia (CLIP) cluster randomised trials.

**Methods:**

The CLIP trials took place in India, Pakistan, and Mozambique, enrolling pregnant women aged 12–49 years between Nov 1, 2014, and Feb 28, 2017. 69 330 pregnant women were enrolled in 44 clusters (36 008 in the 22 intervention clusters and 33 322 in the 22 control clusters). In this secondary analysis of maternal deaths in CLIP, we included women who died in any of the 22 intervention clusters or 22 control clusters. Trained staff administered the WHO 2012 verbal autopsy after maternal deaths. Two physicians (and a third for consensus, if needed) reviewed trial surveillance data and verbal autopsies, and, in intervention clusters, community health worker-led visit data. They determined cause of death according to the WHO International Classification of Diseases-Maternal Mortality (ICD-MM). Verbal autopsies were also analysed by InterVA computer models (versions 4 and 5) to generate cause of death. COMCAT analysis was provided by InterVA-5 and, in India, by physician review of Maternal Newborn Health Registry data. Causes of death and COMCATs assigned by physician review, Inter-VA-4, and InterVA-5 were compared, with agreement assessed with Cohen's κ coefficient.

**Findings:**

Of 61 988 pregnancies with successful follow-up in the CLIP trials, 143 maternal deaths were reported (16 deaths in India, 105 in Pakistan, and 22 in Mozambique). The maternal death rate was 231 (95% CI 193–268) per 100 000 identified pregnancies. Most deaths were attributed to direct maternal causes (rather than indirect or undetermined causes as per ICD-MM classification), with fair to good agreement between physician review and InterVA-4 (κ=0·56 [95% CI 0·43–0·66]) or InterVA-5 (κ=0·44 [0·30–0·57]), and InterVA-4 and InterVA-5 (κ=0·72 [0·60–0·84]). The top three causes of death were the same by physician review, InterVA-4, and InterVA-5 (ICD-MM categories obstetric haemorrhage, non-obstetric complications, and hypertensive disorders); however, attribution of individual patient deaths to obstetric haemorrhage varied more between methods (physician review, 38 [27%] deaths; InterVA-4, 69 [48%] deaths; and InterVA-5, 82 [57%] deaths), than did attribution to non-obstetric causes (physician review, 39 [27%] deaths; InterVA-4, 37 [26%] deaths; and InterVA-5, 28 [20%] deaths) or hypertensive disorders (physician review, 23 [16%] deaths; InterVA-4, 25 [17%] deaths; and InterVA-5, 24 [17%] deaths). Agreement for all nine ICD-MM categories was fair for physician review versus InterVA-4 (κ=0·48 [0·38–0·58]), poor for physician review versus InterVA-5 (κ=0·36 [0·27–0·46]), and good for InterVA-4 versus InterVA-5 (κ=0·69 [0·59–0·79]). The most commonly assigned COMCATs by InterVA-5 were emergencies (68 [48%] of 143 deaths) and health systems (62 [43%] deaths), and by physician review (India only) were health systems (seven [44%] of 16 deaths) and inevitability (five [31%] deaths); agreement between InterVA-5 and physician review (India data only) was poor (κ=0·04 [0·00–0·15]).

**Interpretation:**

Our findings indicate that InterVA-5 is less accurate than InterVA-4 at ascertaining causes and circumstances of maternal death, when compared with physician review. Our results suggest a need to improve the next iteration of InterVA, and for researchers and clinicians to preferentially use InterVA-4 when recording maternal deaths.

**Funding:**

University of British Columbia (grantee of the Bill & Melinda Gates Foundation).

**Research in context**

**Evidence before this study**

Based on our use of the 2012 WHO verbal autopsy and the publication date of the interpreting verbal autopsy model, version 4 (InterVA-4), we searched PubMed from Jan 1, 2012, to March 31, 2020 (updated on Oct 15, 2020), for publications on “maternal death” OR “maternal mortality” AND “verbal autopsy” AND “InterVA-4” OR “InterVA-5” OR “physician review”. We limited our search to publications in English. We identified that, for maternal deaths, agreement on cause of death between physician review and computerised analysis of verbal autopsies with InterVA-4 has often, but not uniformly, been reported to be high, but usually with use of facility records, introducing a high risk of bias compared with population-level data.

**Added value of this study**

To our knowledge, this study is the first to specifically compare physician review with the InterVA-4 and InterVA-5 models for assignment of maternal cause of death. We also included the novel circumstances of mortality categories (COMCATs) component of InterVA-5 and compared these findings with COMCAT analysis by physician review. Although physician review and the InterVA-4 and InterVA-5 algorithms identified the same most common maternal causes of death (obstetric haemorrhage, non-obstetric complications, and hypertensive disorders), we found poor agreement between physician review and InterVA-5 on causes of death in individuals. The InterVA-5 COMCAT results emphasise the importance of quality and continuity of care in facilities, which should be prioritised over care-seeking and transport to health facilities. Our findings are strengthened by a relatively large sample of maternal deaths, in urban and rural settings in south Asia and sub-Saharan Africa, and by use of data from communities with inadequate vital registration systems and the greatest need for computerised cause of death analysis of verbal autopsies.

**Implications of all the available evidence**

Our findings suggest that InterVA-4, and not InterVA-5, should be used to ascertain maternal cause of death and assist in reaching the WHO global standard of registering at least 50% of deaths in communities. Further work is required to refine the InterVA-5 cause of death and COMCAT analyses, and to identify opportunities for improving outcomes in pregnant and post-partum women.

## Introduction

In 2015, more than 300 000 women died from complications during pregnancy or childbirth,^1^ mostly in low-income and middle-income countries in sub-Saharan Africa and south Asia. Although the global maternal mortality rate (MMR) decreased during the Millennium Development Goal era (from 330 deaths per 100 000 livebirths in 2000 to 210 deaths per 100 000 in 2013),^2^ achieving the Sustainable Development Goal MMR of fewer than 70 deaths per 100 000 livebirths by 2030 will require action by communities, health-care providers, and policy makers to address what underlies those deaths. However, the burden, timing, and antecedents of maternal death are incompletely understood, largely due to a scarcity of comprehensive vital registration data.^3^

Various approaches have been developed to understand the health-care journeys of women who have died during pregnancy or post partum. Physician review is effective in improving care and outcomes in settings where records are good and death occurs in health facilities (eg, the UK and Ireland^4^), but this approach is time consuming and challenged by incomplete documentation of all deaths, poor quality record keeping, and fear of retribution and legal action against those involved in the care of women who died. In settings where death registration is absent or incomplete, an alternative approach to understanding maternal deaths is verbal autopsy, a standardised interview with the deceased's next of kin that is designed to explore the woman's symptoms preceding her death, and the circumstances in which her death occurred. Computerised logarithmic probability models have been developed to analyse verbal autopsy responses, to establish reliable temporal and regional estimates of the probable cause of maternal death, with results that were similar to those obtained by physician review.^5^ The interpreting verbal autopsies (InterVA) probabilistic model was developed in 2012 by WHO, and is regularly updated to optimise accuracy in specific circumstances, such as maternal mortality.^6^

In particular, the hypertensive disorders of pregnancy and pre-eclampsia are recognised to be a leading cause of maternal mortality worldwide. The Community-Level Interventions for Pre-eclampsia (CLIP) cluster randomised controlled trials (RCTs) were designed to evaluate whether community engagement and community-level triage, transport, and treatment initiation could reduce all-cause maternal (and perinatal) deaths and morbidity, ascertained in the community, with maternal death evaluated with the WHO verbal autopsy questionnaire (2012 version).^7^ Although all-cause mortality in the CLIP trials did not differ between intervention and control clusters, in this secondary analysis we undertook physician review and InterVA assessments of all maternal deaths. Our aim was to compare agreement between physician review and InterVA methodologies with regard to cause of death and associated circumstances, to inform efforts for improving care.

## Methods

### CLIP trials design and participants

The CLIP trials were independent cluster RCTs in urban and rural India (12 clusters), Pakistan (20 clusters), and Mozambique (12 clusters).^7^ Of the 44 clusters across the three trials, 22 were intervention clusters and 22 were control clusters.

In brief, pregnant women aged 15–49 years (12–49 years in Mozambique) were enrolled after confirmation of pregnancy and provision of informed consent, in India (Nov 1, 2014, to Oct 31, 2016), Pakistan (Jan 1, 2015, to Dec 31, 2016), and Mozambique (Feb 1, 2015, to Feb 28, 2017). In the three CLIP trials, 69 330 pregnant women were enrolled in 44 clusters (36 008 in the 22 intervention clusters and 33 322 in the 22 control clusters), with successful follow-up for 61 988 (89%) pregnancies.^7^ The intervention had two components: community engagement and clinical assessment by community health workers (mobile health-guided early detection); and initial treatment (ie, oral methyldopa for severe hypertension, and intramuscular magnesium sulphate for severe pre-eclampsia) and referral to a facility for hypertensive pregnancies. CLIP visits were recommended at least every 4 weeks before birth, and on post-partum days 3, 7, and 14. In control clusters, women received usual care according to local practice, stated to be blood pressure measurement and proteinuria testing at each antenatal care contact.

The primary outcome was a composite of maternal, fetal, and newborn death and severe morbidity; mortality was measured up to 6 weeks after birth for the mother and 28 days after birth for the neonate. Surveillance data were collected by cross-sectional household surveys (quarterly in Pakistan and every 6 months in Mozambique) or from the Global Network's Maternal Newborn Health (MNH) Registry (in India).^8^ After obtaining individual informed consent, data were collected on baseline individual-level and household-level information, antenatal care, and adverse outcomes up to 6 weeks after birth (for the mother) or 28 days after birth (for the neonate). Data collection tools were modified validated questionnaires translated into local languages. All maternal deaths (and perinatal deaths and maternal and neonatal morbidities) were confirmed by outcome adjudication of trial surveillance data, by in-country teams (primarily doctors) familiar with local language (for interpretation of narratives, if available) and context, but who were masked to cluster type and uninvolved in the women's care. An independent trained team administered the WHO 2012 verbal autopsy^9^ as soon as possible after maternal death, by visiting the deceased woman's household and interviewing a next of kin about health problems, symptoms, and circumstances in the time leading up to the woman's death. In India, Mozambique, and some districts in Pakistan (those with the staffing capacity), interviewees asked next of kin at the end of the structured interview to describe in their own words what had happened to their loved one; these narratives were translated into English and used in physician review.

Data were entered on Android devices and de-identified, and transferred regularly to the University of British Columbia (UBC) CLIP coordinating centre (Vancouver, BC, Canada). Data were uploaded monthly onto the Research Electronic Data Capture server (version 5; Vanderbilt University, Nashville, TN, USA). Data management protocols ensured security (encryption), tracking (user identification numbers and audit trails), and synchronisation between devices within the cluster and with the Research Electronic Data Capture server.

### Procedures

For this secondary analysis, we included women who died in any of the 44 intervention or control clusters, as our primary interest was in measuring the causes and circumstances of maternal death.

In a central process at UBC, for all maternal deaths, two doctors independently reviewed all trial surveillance data and verbal autopsies, and community health worker-led visit data for intervention clusters. The reviewers' experience was in maternal–fetal medicine (PvD), obstetrics (AMA or KA), and obstetric medicine (LAM; also an assessor and chapter writer for the UK and Ireland Confidential Enquiries into Maternal Death and Morbidity). The reviewers were masked to the InterVA-generated cause of death until the cause of death from physician review was established. Cause of death was classified according to the nine categories of the WHO International Classification of Diseases-Maternal Mortality (ICD-MM).^10^ Disagreement was resolved by consensus, involving a third doctor, if necessary. If consensus could not be reached, cause of death was that assigned by two reviewers who agreed. If no two agreed, cause of death was considered undetermined. In India, supplementary data from the MNH Registry was used to facilitate the physician review of circumstances of death.

For all maternal deaths, the 2012 WHO verbal autopsy, a questionnaire specifically designed by WHO for automated processing on the basis of previous validation between physician review and causes of death, was analysed by the InterVA suite of computer models (versions 4 and 5) to generate cause of death categorised according to the ICD-MM. InterVA-4 was available at the time that the CLIP trials were planned and executed; InterVA-5 was included when released during the trials to provide the most useful comparisons. The a priori probabilities for each of malaria and HIV were set as high for Mozambique and low for India and Pakistan. If the InterVA cause of death output for a woman gave more than one possible cause of death, the one with the highest probability was used for comparison with cause of death assigned by physician review.

InterVA-5 was published in 2018, to harmonise with the new WHO verbal autopsy 2016 standard, following updates to the 2012 WHO verbal autopsy based on the processing of more than 650 000 verbal autopsy reports with InterVA-4.^6^ Of relevance to this analysis, InterVA-5 incorporates a novel circumstances of mortality categories (COMCAT) analysis,^11^ with circumstances categorised as traditions, emergencies, recognition, resources, health systems, inevitability, and multiple^11^ (definitions provided in the panel). In India, where additional information was provided by MNH Registry data, clinical care was evaluated as part of the physician review process to ascertain whether improvements in care could be identified that might have made a difference to survival outcome (as opposed to good care, with any improvements in care identified as unlikely to have made a difference to outcome), as undertaken in the UK and Ireland Confidential Enquiries into Maternal Deaths and Morbidity;^4^ the results informed a COMCAT assessment by physician review, in which inevitability was the classification assigned when no improvements in care were identified. We present the InterVA-5 COMCAT assessment of all three trials and physician review-generated assessments of the India trial data. A formal qualitative analysis of verbal autopsy narratives from all three countries is being undertaken and a separate publication is planned.

Panel **Circumstances of mortality categories (COMCAT) definitions11**

• Traditions: traditional practices or beliefs influenced health seeking behaviour and the pathway to death
• Emergencies: sudden, urgent, or unexpected conditions leading to death, which probably precluded life-saving actions
• Recognition: poor recognition or awareness of serious disease, such as symptoms or severity, had a negative influence on health seeking behaviour
• Resources: inability to mobilise and use resources, such as material, transport, or financial, that hindered access to health care
• Health systems: problems in getting health care despite accessing health facilities, such as problems related to admissions, treatments, and medications
• Inevitability: death occurred in circumstances that could not reasonably have been averted, such as in the context of old age or recognised terminal conditions
• Multiple: a combination of the other COMCATs affected the pathway to death, with no single factor predominant at a likelihood of more than 50%

### Statistical analysis

Descriptive analyses were undertaken, overall and by country, to evaluate MMR (maternal deaths per 100 000 livebirths) and maternal death rate (MDR, as the number of maternal deaths per 100 000 identified pregnancies); maternal and pregnancy characteristics at enrolment (baseline) and associated pregnancy outcomes (from trial surveillance); the details of maternal deaths (from trial surveillance and verbal autopsy forms); and causes of death and, for India only, COMCATs, assigned by physician review, InterVA-4, and InterVA-5. For MMR and MDR we calculated 95% Wald-type CIs.

InterVA-4 assignment of hypertensive disorders of pregnancy as a cause of death, as a secondary interest of our analysis, was compared between intervention and control clusters, with multilevel modelling and adjusted odds ratios (ORs) with 95% CIs (fixed effects for maternal age, parity [nulliparous *vs* parous], and basic education, and random effects for country and cluster). Basic education was defined as primary education (country specific) and determined from self-reported number of years of school attended. Mortality was also compared between intervention and control clusters with similar adjusted models (excluding random effects for cluster).

The maternal causes of death assigned by physician review, InterVA-4, and InterVA-5 were compared for overall deaths and by country, according to a three-category classification (as per the ICD-MM) of direct maternal cause, indirect maternal cause, or undetermined; the nine-category ICD-MM classification; and a seven-category COMCAT classification (India data only). Agreement was estimated with Cohen's κ coefficient with 95% CIs; agreement was considered poor for κ values less than 0·40, fair to good for κ values in the range 0·40–0·75, and excellent for κ values greater than 0·75.^12^ Analyses were done with R programming software (version 3.3.2).

The CLIP trials were approved by the UBC research ethics board (H12-03497), and within each country (KLE University [MDC/IECHSR/2013-14/A and ICMR 5/7/859/12-RHN], India; Aga Khan University [2590-Obs-ERC-13], Pakistan; and Centro de Investigação em Saúde da Manhiça [CIBS-CISM/038/14] and Mozambique National Bioethic Committee [219/CNBS/13], Mozambique). The CONSORT and STROBE checklists are in the appendix (pp 3–11).

### Role of the funding source

The funder of the study had no role in study design, data collection, data analysis, data interpretation, or writing of the report.

## Results

Among the 61 988 pregnancies with successful follow-up in the CLIP trials, 143 maternal deaths were reported (16 deaths in India, 105 in Pakistan, and 22 in Mozambique). For 127 (89%) deaths, data were acquired solely in women's communities rather than from facilities. The probability of maternal death did not differ in the intervention clusters (77 deaths) versus control clusters (66 deaths), adjusted for trial setting (country and cluster) and baseline maternal characteristics (age, parity, and basic education; adjusted OR 1·05 [95% CI 0·67–1·64], p=0·84).

From the 143 maternal deaths, we calculated an overall MMR of 253 (95% CI 212–295) per 100 000 livebirths, and an MDR of 231 (95% CI 193–268) per 100 000 identified pregnancies, with the lowest MDR in India (123 [63–183] per 100 000) and the highest in Pakistan (293 [237–349] per 100 000; table 1).

**Table 1:** Baseline maternal characteristics, CLIP intervention, and pregnancy outcomes for 143 maternal deaths in the CLIP trials

		**Total (N=143)**	**India (N=16)**	**Pakistan (N=105)**	**Mozambique (N=22)**
**Incidence of maternal deaths**					
MMR, deaths per 100 000 livebirths		253 (212–295)	145 (74–216)	319 (259–381)	174 (102–247)
MDR, deaths per 100 000 identified pregnancies		231 (193–268)	123 (63–183)	293 (237–349)	167 (97–237)
**Maternal and pregnancy characteristics**					
Maternal age, years		28·0 (24·5–32·0)	21·5 (20·0–24·0)	30·0 (25·0–32·0)	26·5 (22·3–34·0)
Nulliparous		32 (22%)	7 (44%)	19 (18%)	6 (27%)
	Missing	6 (4%)	0	6 (6%)	0
Basic education		37 (26%)	12 (75%)	13 (12%)	12 (55%)
Married		132 (92%)	16 (100%)	105 (100%)	11 (50%)
Gestational age at enrolment, weeks		19·8 (14·6–26·4)	11·2 (8·5–13·5)	21·2 (15·9–26·8)	21·7 (17·5–30·5)
Number of routine antenatal care visits		3 (1–5)	5 (4–6)	2 (1–4)	0 (0–4)
	At least one visit	110 (77%)	16 (100%)	84 (80%)	10 (45%)
	At least four visits	55 (38%)	13 (81%)	35 (33%)	7 (32%)
**Details of maternal deaths**					
Latency between death and verbal autopsy completion, days		62 (33–103)	8 (3–21)	64 (35–100)	158 (47–252)
Respondent for the verbal autopsy					
	Husband	21 (15%)	8 (50%)	10 (10%)	3 (14%)
	Sibling (sister or brother)	22 (15%)	0	19 (18%)	3 (14%)
	Sister in-law or brother-in-law	25 (17%)	2 (13%)	22 (21%)	1 (5%)
	Mother or father	13 (9%)	1 (6%)	8 (8%)	4 (18%)
	Mother in-law or father-in-law	47 (33%)	3 (19%)	41 (39%)*	3 (14%)
	Other (requested to specify)	15 (10%)	2 (13%)†	5 (5%)*	8 (36%)‡
**Antecedent maternal morbidities documented**					
One or more serious end-organ complications		56 (39%)	12 (75%)	38 (36%)	6 (27%)
	Seizure	11 (8%)	5 (31%)	5 (5%)	1 (5%)
	Stroke	14 (10%)	6 (38%)	6 (6%)	2 (9%)
	Coma	10 (7%)	1 (6%)	6 (6%)	3 (14%)
	Antepartum haemorrhage	11 (8%)	1 (6%)	9 (9%)	1 (5%)
	Disseminated intravascular coagulation	5 (3%)	2 (13%)	2 (2%)	1 (5%)
	Sepsis	13 (9%)	2 (13%)	8 (8%)	3 (14%)
	Fistula	0	0	0	0
Life-saving interventions given during pregnancy		40 (28%)	9 (56%)	28 (27%)	3 (14%)
	Cardiopulmonary resuscitation	24 (17%)	3 (19%)	19 (18%)	2 (9%)
	Dialysis	3 (2%)	1 (6%)	2 (2%)	0
	Mechanical ventilation	8 (6%)	3 (19%)	3 (3%)	2 (9%)
	Blood transfusion	22 (15%)	6 (38%)	16 (15%)	0
	Intervention for major post-partum haemorrhage	5 (3%)	2 (13%)	2 (2%)	1 (5%)
	Missing	16 (11%)	0	14 (13%)	2 (9%)
**Location of death**§					
Home		24 (17%)	1 (6%)	19 (18%)	4 (18%)
In transit to facility		16 (11%)	5 (31%)	10 (10%)	1 (5%)
Health facility		103 (72%)	10 (63%)	76 (72%)	17 (77%)
**Timing of death**§					
Antepartum		38 (27%)	5 (31%)	28 (27%)	5 (23%)
Post partum		105 (73%)	11 (69%)	77 (73%)	17 (77%)
**For women who died antepartum**					
Gestational age at death, weeks§		34·5 (30·8–38·3)	30·0 (28·5–33·5)	35·5 (32·3–39·0)	38·0 (28·0–39·0)
**For women who died post partum**					
Gestational age at delivery, weeks		38·0 (34·0–40·0)	37·0 (31·0–41·0)	38·0 (34·5–40·0)	35·0 (34·5–41·0)
Timing of death post partum§					
	<24 h	59/105 (56%)	6/11 (55%)	46/77 (60%)	7/17 (41%)
	1–6 days	17/105 (16%)	2/11 (18%)	12/77 (16%)	3/17 (18%)
	1–6 weeks	24/105 (23%)	3/11 (27%)	15/77 (19%)	6/17 (35%)
	Missing	5/105 (5%)	0	4/77 (5%)	1/17 (6%)
Mode of delivery§					
	Caesarean	38/105 (36%)	4/11 (36%)	26/77 (34%)	8/17 (47%)
	Vaginal birth	67/105 (64%)	7/11 (64%)	51/77 (66%)	9/17 (53%)
Location of birth§					
	Home	16/105 (15%)	1/11 (9%)	11/77 (14%)	4/17 (24%)
	Health facility	89/105 (85%)	10/11 (91%)	66/77 (86%)	13/17 (76%)
	In transit	0	0	0	0
**Pregnancy outcome for all women**					
Stillbirth		28 (20%)	1 (6%)	20 (19%)	7 (32%)
Early neonatal death (≤7 days after birth)		19 (13%)	2 (13%)	14 (13%)	3 (14%)
Late neonatal death (>7 days after birth)		4 (3%)	0	3 (3%)	1 (5%)
Survived follow-up		92 (64%)	13 (81%)	68 (65%)	11 (50%)

**Da**ta are n (%), n/N (%), or median (IQR); values in parentheses for MMR and MDR are 95% CIs. Percentages might not always add to 100% due to rounding. CLIP=Community-Level Interventions for Pre-eclampsia. MMR=maternal mortality ratio. MDR=maternal death rate.

* The other respondents in Pakistan were aunt (n=3), grandparent (n=1), or child (n=1); all of the mother-in-law or father-in-law respondents were mothers-in-law.

† The other respondents in India were grandparent (n=2).

‡ The other respondents in Mozambique were aunt (n=1), child (n=2), child-in-law (n=1), grandparent (n=1), a neighbour (n=1), or a so-called rival (co-spouse or other sexual partner of the father of the pregnancy; n=2).

§ These data were taken from verbal autopsies as the information was not available from trial surveillance reports.

Women who died were young, ranging in median age from 21·5 years (IQR 20·0–24·0) in India to 30·0 years (25·0–32·0) in Pakistan (table 1). Most women were parous, particularly in Pakistan. A basic level of education was reported by most women in India, more than half in Mozambique, and a minority in Pakistan. All women were married in India and Pakistan, but only half were married in Mozambique. Generally, women who died in India had attended at least one antenatal care appointment in the first trimester (gestational weeks 1–12; ten [63%] of 16 women) and most had received at least four routine antenatal care visits. In Pakistan and Mozambique, women who died had attended their first appointment at a median of 21–22 weeks' gestation (middle of their second trimester), and only about a third had received four or more antenatal care visits. Few women had attended their first appointment in the first trimester (ten [10%] of 105 women in Pakistan and two [9%] of 22 in Mozambique).

Details of maternal deaths were recorded by verbal autopsy as early as one week (median 8 days [IQR 3–21]) after death in India, to more than 5 months (158 days [47–252]) after death in Mozambique (table 1). The verbal autopsy respondent differed by country, and was most often the husband (widower) in India and the husband's parents (parents-in-law) in Pakistan, with a wide variety of respondents in Mozambique.

The frequency with which women had antecedent maternal morbidity varied by country. Most of the women who died in India had a documented serious end-organ complication, followed by around a third of the women in Pakistan, and about a quarter of the women in Mozambique (table 1). In addition, morbidity was defined by having received a potentially life-saving intervention, typically cardiopulmonary resuscitation or blood transfusion.

Most women died in a health facility, although about a third died in transit to a facility in India (table 1). About a quarter of women died antepartum and, by definition, undelivered. Based on median gestational age at death in each country, antepartum deaths typically occurred early in the third trimester (India), late preterm (Pakistan), or at term (Mozambique). About three-quarters of women died post partum, either after late preterm delivery (Mozambique) or at term (India and Pakistan). These women most frequently died within 24 h post partum, although many died more than 1 week after birth. Mode of delivery was usually vaginal and at a health facility. Among all women who died, stillbirth was frequent, particularly in Pakistan and Mozambique, and a fifth of babies born alive subsequently died, usually within the first 7 days after birth.

Cause of death assigned by physician review was informed by next-of-kin narratives (in addition to the structured verbal autopsy interview) for all deaths in India and Mozambique, and 55 (52%) of 105 deaths in Pakistan. In the physician review and InterVA assignment of total maternal deaths (n=143) as having direct maternal cause, indirect maternal cause, or undetermined, deaths were most often attributed to direct maternal causes (table 2). This was true overall and by country, with the exception of physician review assignments in Mozambique, where slightly more than half of deaths (12 [55%] of 22) were attributed to indirect maternal causes. Expectedly, these deaths in Mozambique were attributed by physician review to non-obstetric complications among the nine categories of the ICD-MM, related primarily to infectious diseases (n=9; most commonly HIV [n=2] and malaria [n=4]), or cardiac disease (n=3; appendix pp 12–14). For the three-category classification of maternal cause of death (direct maternal, indirect maternal, or undetermined), we observed fair to good agreement between physician review and either InterVA-4 (κ=0·56 [95% CI 0·43–0·66]) or InterVA-5 (κ=0·44 [0·30–0·57]), and between InterVA-4 and InterVA-5 (κ=0·72 [0·60–0·84]; table 3).

**Table 2:** Maternal causes of death as determined by InterVA and physician review for overall deaths (N=143)

			**Physician review**	**InterVA-4**	**InterVA-5**
**Three-category classification (ICD-MM)**					
Direct maternal cause			88 (62%)	100 (70%)	110 (77%)
Indirect maternal cause			39 (27%)	37 (26%)	28 (20%)
Undetermined cause			16 (11%)	6 (4%)	5 (3%)
**Nine-category classification (ICD-MM)**					
(1) Abortive outcomes			1 (1%)	0	1 (1%)
(2) Hypertensive disorders			23 (16%)	25 (17%)	24 (17%)
(3) Obstetric haemorrhage			38 (27%)	69 (48%)	82 (57%)
(4) Pregnancy-related infection			6 (4%)	2 (1%)	0
(5) Other obstetric complications			16 (11%)	4 (3%)	3 (2%)
	Venous thromboembolism		8/16	0	0
	Uterine inversion or rupture		2/16	0	1/3
	Suicide		2/16	1/4	1/3
	Obstructed labour		0	3/4	1/3
	Other*		4/16	0	0
(6) Unanticipated complications of clinical management			4 (3%)	0	0
(7) Non-obstetric complications			39 (27%)	37 (26%)	28 (20%)
	Infectious disease		26/39	28/37	20/28
		Respiratory	9	11	9
		Gastrointestinal	3	1	0
		Malaria	5	4	1
		HIV	2	8	7
		Tuberculosis	2	3	1
		Other†	5	1	2
	Cardiac disease		10/39	3/37	4/28
	Liver disease		2/39	3/37	2/28
	Other‡		1/39	3/37	2/28
(8) Unknown or undetermined			16 (11%)	6 (4%)	4 (3%)
(9) Coincidental causes			0	0	1 (1%)
**COMCAT**§					
Traditions			0¶	NA	1/143 (1%)
Emergencies			2/16 (13%)¶	NA	68/143 (48%)
Recognition			2/16 (13%)¶	NA	1/143 (1%)
Resources			0	NA	9/143 (6%)
Health systems			7/16 (44%)¶	NA	62/143 (43%)
Inevitability			5/16 (31%)¶	NA	0
Multiple			0¶	NA	2/143 (1%)

**Da**ta are numbers of women and percentages. Percentages might not always add to 100% due to rounding. COMCAT=circumstances of mortality categories. ICD-MM=International Classification of Diseases-Maternal Mortality. InterVA=interpreting verbal autopsy. NA=not applicable.

* Other obstetric complications were amniotic fluid embolism (n=1), peripartum cardiomyopathy (n=1), complications of intrauterine fetal demise (n=1), and disseminated intravascular coagulation (n=1).

† Other infections were meningitis (n=2), tetanus (n=1), hepatitis (n=1), measles (n=1), and infections not otherwise specified (n=3).

‡ Other non-obstetric causes were stroke (n=1), breast neoplasm (n=1), asthma (n=2), and chronic obstructive pulmonary disease (n=2).

§ COMCAT definitions are provided in the panel.

¶ India only.

**Table 3:** Cohen's κ statistics for agreement between methods of assigned maternal cause of death (N=143)

	κ (95% CI)*
**Three-category classification (ICD-MM)**	
Physician review versus InterVA-4	0·56 (0·43–0·66)
Physician review versus InterVA-5	0·44 (0·30–0·57)
InterVA-4 versus InterVA-5	0·72 (0·60–0·84)
**Nine-category classification (ICD-MM)**	
Physician review versus InterVA-4	0·48 (0·38–0·58)
Physician review versus InterVA-5	0·36 (0·27–0·46)
InterVA-4 versus InterVA-5	0·69 (0·59–0·79)

ICD-MM=International Classification of Diseases-Maternal Mortality. InterVA=interpreting verbal autopsy.

* A κ statistic of less than 0·40 was considered poor agreement, 0·40–0·75 fair to good agreement, and greater than 0·75 excellent agreement.

In the ICD-MM classification of specific cause of death, the three top causes for overall deaths were the same by physician review, InterVA-4, and InterVA-5 (figure). These were obstetric haemorrhage (ICD-MM category 3), followed by non-obstetric complications (ICD-MM category 7), and hypertensive disorders (ICD-MM category 2). Hypertensive disorders as a cause of death (by InterVA-4) did not differ in the intervention clusters (13 [17%] of 77 deaths) versus the control clusters (12 [18%] of 66 deaths; OR 0·93 [95% CI 0·46–1·89]; p=0·84). Attribution to obstetric haemorrhage varied more between methods (physician review, 38 [27%] deaths; InterVA-4, 69 [48%] deaths; and InterVA-5, 82 [57%] deaths) than did attribution to non-obstetric causes (physician review, 39 [27%] deaths; InterVA-4, 37 [26%] deaths; and InterVA-5, 28 [20%] deaths), or hypertensive disorders (physician review, 23 [16%] deaths; InterVA-4, 25 [17%] deaths; and InterVA-5, 24 [17%] deaths; table 2, figure). Few maternal deaths (<5% and often <1%) were attributed to pregnancy-related infection, abortive outcomes, unanticipated complications of management, or coincidental causes (figure, table 2). A small proportion of all maternal causes of death were unknown or undetermined (16 [11%] deaths assigned by physician review, six [4%] by InterVA-4, and four [3%] by InterVA-5). Agreement between the cause of death methods for the nine ICD-MM categories was fair for physician review versus InterVA-4, poor for physician review versus InterVA-5, and good for InterVA-4 versus InterVA-5 (table 3); similar results were observed by country (appendix p 15). Physician review identified that in cases of shock, women could be assigned by InterVA as having obstetric haemorrhage without any evidence of bleeding (31 ([22%] and 44 [31%] additional cases of obstetric haemorrhage by InterVA-4 and InterVA-5, respectively). By physician review, these cases were distributed between other causes of death excluding hypertension.

**Figure:**
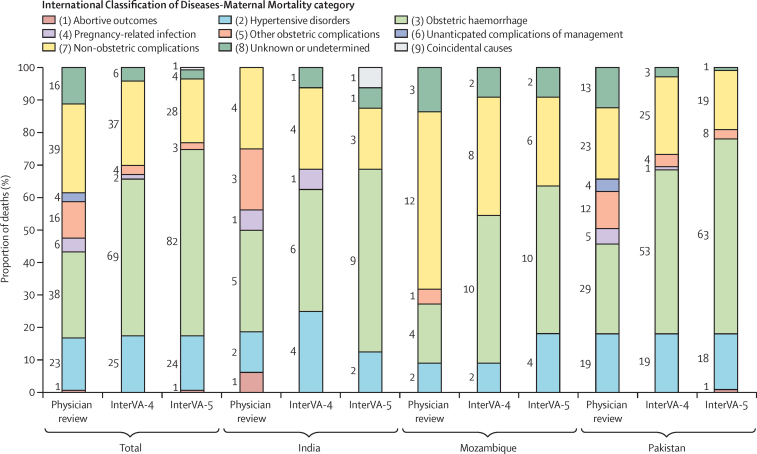
Maternal causes of death according to physician review, InterVA-4, and InterVA-5 (N=143) Number of women are presented for each cause. InterVA=interpreting verbal autopsy.

For circumstances of death, InterVA-5 most commonly assigned the COMCAT classifications of emergencies (68 [48%] of 143 deaths) and health systems (62 [43%] deaths); whereas, few maternal deaths were assigned to traditions, recognition, or multiple circumstances. By physician review (possible only in India), health systems (seven [44%] of 16 deaths) and inevitability (five [31%] deaths) were the most common circumstances of death (table 2). Agreement between physician review and InterVA-5 for COMCAT (India data only) was poor (κ=0·04 [95% CI 0·00–0·15).

During physician review of maternal deaths in India, distinguishing between the COMCATs of emergencies and inevitability was difficult without direct review of health records to understand quality of care. Physician review of summary data in the MNH Registry identified six of the 16 maternal deaths for which improvements in care might have made a difference to outcome. Five were classified under health systems circumstances, related to suboptimal facility care (n=3 for hypertensive pregnancies, either failure to administer antihypertensives for severe hypertension [n=2] or to initiate birth for pre-eclampsia at term [n=1]; and n=2 for obstetric haemorrhage when no intervention for the haemorrhage was offered). In one case of cardiovascular collapse in transit to facility (deemed an emergency), no cardiopulmonary resuscitation was attempted.

Although quality of care could not be assessed formally in Pakistan or Mozambique due to the scarcity of detailed information from health facilities, the narratives from next of kin did highlight some of the health systems failures identified by the InterVA-5 COMCAT analysis of deaths overall, and by physician review in India. Reviewers noted several instances in which the woman's relatives were asked to arrange blood products for transfusion, even during resuscitation for obstetric haemorrhage. In addition, reviewers noted several instances in which the woman was ill and attended a health facility (private or public), but they were deemed too ill for that facility, refused assessment or treatment, and advised to seek help at another facility, without transport assistance. Publication of the complete verbal autopsy analysis is planned.

## Discussion

For the 143 maternal deaths (MMR 253 [95% CI 212–295] per 100 000 livebirths) in the CLIP trials in India, Pakistan, and Mozambique, we found that physician review and the computer algorithms of InterVA-4 and InterVA-5 had high public health equivalence, revealing the same top three maternal causes of death: obstetric haemorrhage, non-obstetric causes, and hypertensive disorders of pregnancy. However, attribution to each cause of death varied by method; of the three methods, InterVA-5 was most likely to assign obstetric haemorrhage and least likely to assign non-obstetric causes as a cause of death. Agreement for physician review versus InterVA-4 was better than for physician review versus InterVA-5. Both the physician review and InterVA-5 COMCAT analyses identified health systems circumstances as underlying more than 40% of maternal deaths, but physician review also commonly identified inevitability of circumstance, whereas emergency circumstances were identified the most by InterVA-5.

Our findings suggest that for public health planning and policy aimed at reducing maternal death, InterVA-4 is a reasonable alternative to physician review; however, obstetric haemorrhage might be over-represented, particularly if women show signs of shock without evidence of bleeding. Physician review was informed by next-of-kin narratives; developments in software interpretation of free text might lead to further improvements in InterVA algorithm performance. InterVA-5 performed poorly for maternal cause of death, particularly in identifying non-obstetric causes. This finding might be increasingly relevant as countries move through stages of obstetric transition and indirect causes of death increase in frequency.^13^

The novel InterVA-5 COMCAT analysis of maternal deaths did not highlight delays in care (in obstetric care-seeking or in reaching an appropriate obstetric facility, commonly referred to as first and second delays, respectively^14^); traditions, recognition, and resources were indicated to be unimportant for most women in the events leading to their deaths. Furthermore, few women were identified to have multiple underlying circumstances. These insights emphasise the importance of delays in receiving quality care (the so-called third delay^14^), especially in continuity of care, as being modifiable contributors to maternal deaths; if addressed, outcomes should improve. Additionally, our findings suggest that for maternal death specifically, further work might be required to distinguish between emergencies (for which outcomes would be avoidable only through prevention, rather than treatment) and inevitability (particularly for women offered good care who, nevertheless, did not avoid death).

To our knowledge, this study provides the first comparison of maternal causes of death by physician review, InterVA-4, and InterVA-5, and of COMCAT analyses by physician review and InterVA-5. One published comparison of InterVA-4 and InterVA-5 focused on HIV and excluded maternal deaths.^15^ A second publication evaluated the causes of all deaths in adults and children, comparing physician-assigned cause of death at tertiary hospitals involved in final care with InterVA-4 and InterVA-5, which found high concordance (eg, InterVA-5 correlation coefficient of 0·86 [0·79–0·93] for adults).^6^

InterVA-4 has been compared with physician review for deaths in general^16^, ^17^, ^18^, ^19^ and for maternal death specifically.^20^ In Africa and Asia among adults or the general population, concordance between physician review and InterVA-4 has been variably reported, as high (≥80%)^17^, ^18^ or not.^16^, ^19^, ^21^ Among 86 maternal deaths analysed at the health-facility level in Malawi, fair to good agreement was reported between an expert physician review panel and InterVA-4 for ICD-MM categories 1–7 (Cohen's κ=0·66), although the expert physician review panel assigned cause of death as unspecified more often than both InterVA-4 and InterVA-5.^20^ In that analysis, the top four assigned direct maternal causes of death for expert physician review and InterVA-4 were obstetric haemorrhage (34% *vs* 30%), pregnancy-related infections (14% *vs* 17%), hypertensive disorders (12% *vs* 16%), and pregnancy with abortive outcomes (14% *vs* 15%). Similar to the Malawi study, agreement between physician review and InterVA-4 in our analysis was fair to good, and physician review identified unknown or indeterminate cause of death more frequently than InterVA-4; however, our κ value was lower (0·48 *vs* 0·66^20^), which might have been due to our assessment of deaths in three countries (rather than one), or that most of our data (89%) for physician review was from the community (rather than from a single health facility).

Strengths of our study include a relatively large sample of deaths, representation of urban and rural settings in south Asia and sub-Saharan Africa, and use of data from communities, making our results generalisable to where there is the greatest need for computerised cause of death analysis of verbal autopsies. We compared physician review and both InterVA-4 and InterVA-5 computerised algorithms for cause of death and COMCAT.

Limitations include those of the verbal autopsy itself, which has questions lacking clarity of purpose, such as the needed or received designation for medication, rather than needed but unavailable or not received, meaning the possibility for improvements in care cannot be determined. Physician review was done by a central team familiar with local contexts, but from none of the CLIP countries. Social factors were considered in this review; however, a social autopsy or diagnosis was not done, based on availability of resources. A verbal autopsy narrative was available for about two-thirds of maternal deaths in Pakistan (65% of deaths overall), and narratives in Mozambique were limited in detail. Although CLIP data forms were available for physician review, the lack of comprehensive, detailed verbal autopsy narratives for all deaths might have compromised cause of death assignment in Pakistan and Mozambique, where quality of care could not be evaluated. The lack of detailed verbal autopsy reports was compounded by limited detail in women's health records in these settings. The between-country differences in respondents and verbal autopsy timing might have had an effect on responses, but the top causes of death and proportion of direct maternal deaths were similar; we had insufficient power to examine these differences by country. Finally, we were not able to identify how weights in the probabilistic model for InterVA-5 differed from InterVA-4, because neither demographic health survey data nor health facility records were available to us.

Our findings suggest that InterVA-4, not InterVA-5, should be used to ascertain maternal cause of death, particularly given the global rise in non-obstetric maternal deaths,^22^ and to assist in reaching WHO's global standard of registering at least 50% of deaths in communities.^23^ Further work is required to refine InterVA-5 regarding assignment of obstetric haemorrhage and non-obstetric causes of death and COMCAT analysis, and to identify opportunities for improving maternal outcomes.

## Data sharing

The data on which the manuscript is based are freely available without restrictions from the CLIP Trials Data Access Committee, who can be contacted at PRE-EMPT@cw.bc.ca, as referenced on the website for the Pregnancy Evidence, Monitoring, Partnerships and Treatment (PRE-EMPT) initiative. The full CLIP Trials Data Sharing Statement is provided in the appendix (pp 16–17).

## Declaration of interests

We declare no competing interests.

## References

[1] UN Statistics Division (2018). The Sustainable Development Goals report 2018. https://unstats.un.org/sdgs/report/2018/overview/.

[2] UN (2015). The Millennium Development Goals report 2015. https://www.un.org/millenniumgoals/2015_MDG_Report/pdf/MDG%202015%20rev%20(July%201).pdf.

[3] Lo S, Horton R (2015). Everyone counts—so count everyone. Lancet.

[4] Knight M, Bunch K, Tuffnell D, on behalf of MBRRACE-UK (2019). Saving lives, improving mothers' care: lessons learned to inform maternity care from the UK and Ireland Confidential Enquiries into Maternal Deaths and Morbidity 2015–17.

[5] Byass P, Fottrell E, Dao LH (2006). Refining a probabilistic model for interpreting verbal autopsy data. Scand J Public Health.

[6] Byass P, Hussain-Alkhateeb L, D'Ambruoso L (2019). An integrated approach to processing WHO-2016 verbal autopsy data: the InterVA-5 model. BMC Med.

[7] von Dadelszen P, Bhutta ZA, Sharma S (2020). The Community-Level Interventions for Pre-eclampsia (CLIP) cluster randomised trials in Mozambique, Pakistan, and India: an individual participant-level meta-analysis. Lancet.

[8] Goudar SS, Carlo WA, McClure EM (2012). The Maternal and Newborn Health Registry study of the Global Network for Women's and Children's Health Research. Int J Gynaecol Obstet.

[9] WHO (2012). Verbal autopsy standards: the 2012 WHO verbal autopsy instrument. https://wwwwhoint/healthinfo/statistics/WHO_VA_2012_RC1_Instrumentpdf?ua=1.

[10] WHO (2012). The WHO application of ICD-10 to deaths during pregnancy, childbirth and puerperium: ICD MM. https://www.who.int/reproductivehealth/publications/monitoring/9789241548458/en/.

[11] Hussain-Alkhateeb L, D'Ambruoso L, Tollman S (2019). Enhancing the value of mortality data for health systems: adding circumstances of mortality categories (COMCATs) to deaths investigated by verbal autopsy. Glob Health Action.

[12] Cohen J (1960). A coefficient of agreement for nominal scales. Educ Psychol Meas.

[13] Souza JP, Tunçalp Ö, Vogel JP (2014). Obstetric transition: the pathway towards ending preventable maternal deaths. BJOG.

[14] Thaddeus S, Maine D (1994). Too far to walk: maternal mortality in context. Soc Sci Med.

[15] Karat AS, Maraba N, Tlali M (2018). Performance of verbal autopsy methods in estimating HIV-associated mortality among adults in South Africa. BMJ Glob Health.

[16] Murray CJ, Lozano R, Flaxman AD (2014). Using verbal autopsy to measure causes of death: the comparative performance of existing methods. BMC Med.

[17] Jha P, Kumar D, Dikshit R (2019). Automated versus physician assignment of cause of death for verbal autopsies: randomized trial of 9374 deaths in 117 villages in India. BMC Med.

[18] Byass P, Herbst K, Fottrell E (2015). Comparing verbal autopsy cause of death findings as determined by physician coding and probabilistic modelling: a public health analysis of 54 000 deaths in Africa and Asia. J Glob Health.

[19] Weldearegawi B, Melaku YA, Dinant GJ, Spigt M (2015). How much do the physician review and InterVA model agree in determining causes of death? A comparative analysis of deaths in rural Ethiopia. BMC Public Health.

[20] Mgawadere F, Unkels R, van den Broek N (2016). Assigning cause of maternal death: a comparison of findings by a facility-based review team, an expert panel using the new ICD-MM cause classification and a computer-based program (InterVA-4). BJOG.

[21] Desai N, Aleksandrowicz L, Miasnikof P (2014). Performance of four computer-coded verbal autopsy methods for cause of death assignment compared with physician coding on 24 000 deaths in low- and middle-income countries. BMC Med.

[22] Kassebaum NJ, Bertozzi-Villa A, Coggeshall MS (2014). Global, regional, and national levels and causes of maternal mortality during 1990–2013: a systematic analysis for the Global Burden of Disease Study 2013. Lancet.

[23] de Savigny D, Riley I, Chandramohan D (2017). Integrating community-based verbal autopsy into civil registration and vital statistics (CRVS): system-level considerations. Glob Health Action.

