# Supplementary material for: Causes and circumstances of maternal death: a secondary analysis of the Community-Level Interventions for Pre-eclampsia (CLIP) trials cohort
Source: Lancet Glob Health. 2021 Jul 29;9(9):e1242–51. doi: 10.1016/S2214-109X(21)00263-1 (PMC8370879; doi:10.1016/S2214-109X(21)00263-1)
Supplement: Supplementary appendix [file mmc1.pdf]

THE LANCET
Global Health

Supplementary appendix

This appendix formed part of the original submission and has been peer reviewed. We post it as supplied by the authors.

Supplement to: Aukes AM, Arion K, Bone JN, et al. Causes and circumstances of maternal death: a secondary analysis of the Community-Level Interventions for Pre-eclampsia (CLIP) trials cohort. *Lancet Glob Health* 2021; published online July 29. http://dx.doi.org/10.1016/S2214-109X(21)00263-1.

APPENDIX

Table/Text	Title	Page number
S1	The CLIP (Community-Level interventions in Pre-eclampsia) Trials Study Group	2
S2	CONSORT Checklist – Extension for Cluster Trials	3
S3	STROBE Checklist	7
S4	Cause of death by country, as determined by InterVA and Physician's review (PR) for 143 maternal deaths in the CLIP trials	12
S5	Kappa statistics by country, for measurement of agreement between methods of assigned maternal COD for the 143 maternal deaths in the CLIP trials	15
S6	Data Sharing Statement	16

Table S1: The CLIP Trials Study Group

**CLIP Trials Working Group**
Esperança Sevene, Eusébio Macete, Khátia Munguambe, Charfudin Sacoor, Anifa Vala, Helena Boene, Felizarda Amose, Rosa Pires, Zefanias Nhamirre, Marta Macamo, Rogério Chiaú, Analisa Matavele, Faustino Vilanculo, Ariel Nhancolo, Silvestre Cutana, Ernesto Mandlate, Salésio Macuacua, Cassimo Bique, Sibone Mocumbi, Emília Gonçálves, Sónia Maculuve, Ana Ilda Biz, Dulce Mulungo, Orvalho Augusto, Paulo Filimone, Vivalde Nobela, Corsino Tchavana, Cláudio Nkumbula
Rahat Qureshi, Zulfiqar A Bhutta, Zahra Hoodbhoy, Farrukh Raza, Sana Sheikh, Javed Memon, Imran Ahmed, Amjad Hussain
Mrutunjaya B Bellad, Umesh S Charantimath, Shivaprasad S Goudar, Geetanjali M Katageri, Avinash J Kavi, Amit P Revankar, Ashalata A Mallapur, Umesh Y Ramdurg, Shashidhar G Bannale, Vaibhav B Dhamanekar, Geetanjali I Mungarwadi, Narayan V Honnungar, Bhalachandra S Kodkany, Anjali M Joshi, Uday S Kudachi, Sphoorthi S Mastiholi, Chandrappa C Karadiguddi, Gudadayya S Kengapur, Namdev A Kamble, Keval S Chougala
Jeffrey N Bone, Dustin T Dunsmuir, Sharla K Drebit, Chirag Kariya, Mai-Lei Woo Kinshella, Tang Lee, Jing Li, Mansun Lui, Beth A Payne, Diane Sawchuck, Sumedha Sharma, Domena K Tu, Marianne Vidler, Ugochi V·Ukah, Laura A Magee, Peter von Dadelszen
**CLIP Trial Steering Committee**
J Mark Ansermino, Ana Pilar Betrán, Richard Derman, Shafik Dharamsi, France Donnay, Sharla Drebit, Guy Dumont, Susheela M Engelbrecht, Veronique Fillipi, Tabassum Firoz, William Grobman, Marian Knight, Ana Langer, Simon Lewin, Gwyneth Lewis, Craig Mitton, Nadine Schuurman, Andrew Shennan, Joel Singer, Jim Thornton, Hubert Wong
**CLIP Trial Executive Committee**
Olalekan O Adetoro, Mrutunjaya M Bellad, Zulfiqar A Bhutta, Peter von Dadelszen, Shivaprasad S Goudar, Laura A Magee, Ashalata A Mallapur, Khátia Munguambe, Beth A Payne, Rahat Qureshi, Charfudin Sacoor, Esperança Sevene, Sumedha Sharma, John O Sotunsa, Marianne Vidler
**CLIP Data Safety and Monitoring Board (DSMB)**
Romano Nkumbwa Byaruhanga, Brian Darlow, Eileen Hutton, Mario Merialdi, Lehana Thabane

Table S2: CONSORT Checklist – Extension for Cluster Trials

Section/Topic	Item No	Standard Checklist item	Extension for cluster designs	Page No *
**Title and abstract**				
	1a	Identification as a randomised trial in the title	Identification as a cluster randomised trial in the title	N/A, this is a secondary analysis. This information is reported in the CLIP Trial primary publications.
	1b	Structured summary of trial design, methods, results, and conclusions (for specific guidance see CONSORT for abstracts)	See table 2	3
**Introduction**				
**Background and objectives**	2a	Scientific background and explanation of rationale	Rationale for using a cluster design	4
	2b	Specific objectives or hypotheses	Whether objectives pertain to the the cluster level, the individual participant level or both	4
**Methods**				
**Trial design**	3a	Description of trial design (such as parallel, factorial) including allocation ratio	Definition of cluster and description of how the design features apply to the clusters	5
	3b	Important changes to methods after trial commencement (such as eligibility criteria), with reasons		N/A
**Participants**	4a	Eligibility criteria for participants	Eligibility criteria for clusters	5
	4b	Settings and locations where the data were collected		5
**Interventions**	5	The interventions for each group with sufficient details to allow replication, including how and when they were actually administered	Whether interventions pertain to the cluster level, the individual participant level or both	5
**Outcomes**	6a	Completely defined pre-specified primary and secondary outcome measures, including how and when they were assessed	Whether outcome measures pertain to the cluster level, the individual participant level or both	5
	6b	Any changes to trial outcomes after the trial commenced, with reasons		N/A
**Sample size**	7a	How sample size was determined	Method of calculation, number of clusters(s) (and whether equal or unequal cluster sizes are assumed), cluster size, a coefficient of intracluster	See Supplementary Appendix (Text S1 and S2)

			correlation (ICC or *k*), and an indication of its uncertainty	
	7b	When applicable, explanation of any interim analyses and stopping guidelines		See Supplementary Appendix (Text S4 and S5)
**Randomisation:**				
**Sequence generation**	8a	Method used to generate the random allocation sequence		See Supplementary Appendix (Text S1 and S2)
	8b	Type of randomisation; details of any restriction (such as blocking and block size)	Details of stratification or matching if used	See Supplementary Appendix (Text S1 and S2)
**Allocation concealment mechanism**	9	Mechanism used to implement the random allocation sequence (such as sequentially numbered containers), describing any steps taken to conceal the sequence until interventions were assigned	Specification that allocation was based on clusters rather than individuals and whether allocation concealment (if any) was at the cluster level, the individual participant level or both	See Supplementary Appendix (Text S1 and S2)
**Implementation**	10	Who generated the random allocation sequence, who enrolled participants, and who assigned participants to interventions	Replace by 10a, 10b and 10c	
	10a		Who generated the random allocation sequence, who enrolled clusters, and who assigned clusters to interventions	See Supplementary Appendix (Text S1 and S2)
	10b		Mechanism by which individual participants were included in clusters for the purposes of the trial (such as complete enumeration, random sampling)	Appendix (Text S1, S2, S4, and S5)
	10c		From whom consent was sought (representatives of the cluster, or individual cluster members, or both), and whether consent was sought before or after randomisation	Appendix (Text S1 and S2)
**Blinding**	11a	If done, who was blinded after assignment to interventions (for example, participants, care providers, those assessing outcomes) and how		5
	11b	If relevant, description of the similarity of interventions		5
**Statistical methods**	12a	Statistical methods used to compare groups for primary and secondary outcomes	How clustering was taken into account	6; See Supplementary Appendix (Text S1, S2, S4 and S5)

	12b	Methods for additional analyses, such as subgroup analyses and adjusted analyses		6; See Supplementary Appendix (Text S4 and S5)
**Results**				
**Participant flow (a diagram is strongly recommended)**	13a	For each group, the numbers of participants who were randomly assigned, received intended treatment, and were analysed for the primary outcome	For each group, the numbers of clusters that were randomly assigned, received intended treatment, and were analysed for the primary outcome	7
	13b	For each group, losses and exclusions after randomisation, together with reasons	For each group, losses and exclusions for both clusters and individual cluster members	7
**Recruitment**	14a	Dates defining the periods of recruitment and follow-up		5
	14b	Why the trial ended or was stopped		N/A
**Baseline data**	15	A table showing baseline demographic and clinical characteristics for each group	Baseline characteristics for the individual and cluster levels as applicable for each group	5
**Numbers analysed**	16	For each group, number of participants (denominator) included in each analysis and whether the analysis was by original assigned groups	For each group, number of clusters included in each analysis	6
**Outcomes and estimation**	17a	For each primary and secondary outcome, results for each group, and the estimated effect size and its precision (such as 95% confidence interval)	Results at the individual or cluster level as applicable and a coefficient of intracluster correlation (ICC or k) for each primary outcome	7-8 (Table 3, S4)
	17b	For binary outcomes, presentation of both absolute and relative effect sizes is recommended		N/A
**Ancillary analyses**	18	Results of any other analyses performed, including subgroup analyses and adjusted analyses, distinguishing pre-specified from exploratory		N/A
**Harms**	19	All important harms or unintended effects in each group (for specific guidance see CONSORT for harms)		5
**Discussion**				
**Limitations**	20	Trial limitations, addressing sources of potential bias, imprecision, and, if relevant, multiplicity of analyses		10

**Generalisability**	21	Generalisability (external validity, applicability) of the trial findings	Generalisability to clusters and/or individual participants (as relevant)	9
**Interpretation**	22	Interpretation consistent with results, balancing benefits and harms, and considering other relevant evidence		9-10
**Other information**				
**Registration**	23	Registration number and name of trial registry		3, 5
**Protocol**	24	Where the full trial protocol can be accessed, if available		See Supplementary Appendix (Text S2)
**Funding**	25	Sources of funding and other support (such as supply of drugs), role of funders		3, 10

** Note: page numbers optional depending on journal requirements*

Table S3: STROBE checklist

	Item	Recommendation	Reported on manuscript page
**Title and abstract**			
	1	(a) Indicate the study's design with a commonly used term in the title or the abstract	3
		(b) Provide in the abstract an informative and balanced summary of what was done and what was found	3
**Introduction**			
Background/rationale	2	Explain the scientific background and rationale for the investigation being reported	4
Objectives	3	State specific objectives, including any prespecified hypotheses	4
**Methods**			
Study design	4	Present key elements of study design early in the paper	5
Setting	5	Describe the setting, locations, and relevant dates, including periods of recruitment, exposure, follow-up, and data collection	5
Participants	6	(a) *Cohort study*—give the eligibility criteria, and the sources and methods of selection of participants. Describe methods of follow-up	
		Case-control study—give the eligibility criteria, and the sources and methods of case ascertainment and control selection. Give the	

	Item	Recommendation	Reported on manuscript page
		rationale for the choice of cases and controls	
		Cross-sectional study—give the eligibility criteria, and the sources and methods of selection of participants	5
		(b) *Cohort study*—for matched studies, give matching criteria and number of exposed and unexposed	
		Case-control study—for matched studies, give matching criteria and the number of controls per case	
Variables	7	Clearly define all outcomes, exposures, predictors, potential confounders, and effect modifiers. Give diagnostic criteria, if applicable	5
Data sources/measurement	8*	For each variable of interest give sources of data and details of methods of assessment (measurement). Describe comparability of assessment methods if there is more than one group	5-6
Bias	9	Describe any efforts to address potential sources of bias	5
Study size	10	Explain how the study size was arrived at	5
Quantitative variables	11	Explain how quantitative variables were handled in the analyses. If applicable, describe which groupings were chosen, and why	6

	Item	Recommendation	Reported on manuscript page
Statistical methods	12	(a) Describe all statistical methods, including those used to control for confounding	6
		(b) Describe any methods used to examine subgroups and interactions	6
		(c) Explain how missing data were addressed	NA
		(d) *Cohort study*—if applicable, explain how loss to follow-up was addressed	
		Case-control study—if applicable, explain how matching of cases and controls was addressed	
		Cross-sectional study—if applicable, describe analytical methods taking account of sampling strategy	
		(e) Describe any sensitivity analyses	NA
**Results**			
Participants	13*	(a) Report the numbers of individuals at each stage of the study—eg, numbers potentially eligible, examined for eligibility, confirmed eligible, included in the study, completing follow-up, and analysed	7
		(b) Give reasons for non-participation at each stage	N/A
		(c) Consider use of a flow diagram	N/A

	Item	Recommendation	Reported on manuscript page
Descriptive data	14*	(a) Give characteristics of study participants (eg, demographic, clinical, social) and information on exposures and potential confounders	7
		(b) Indicate the number of participants with missing data for each variable of interest	7
		(c) *Cohort study*—summarise follow-up time (eg, average and total amount)	
Outcome data	15*	*Cohort study*—report numbers of outcome events or summary measures over time	
		Case-control study—report numbers in each exposure category, or summary measures of exposure	
		Cross-sectional study—report numbers of outcome events or summary measures	7-8
Main results	16	(a) Give unadjusted estimates and, if applicable, confounder-adjusted estimates and their precision (eg, 95% confidence interval). Make clear which confounders were adjusted for and why they were included	7
		(b) Report category boundaries when continuous variables were categorised	N/A
		(c) If relevant, consider translating estimates of relative risk into absolute risk for a meaningful time period	N/A

	Item	Recommendation	Reported on manuscript page
Other analyses	17	Report other analyses done—eg, analyses of subgroups and interactions, and sensitivity analyses	8
**Discussion**			
Key results	18	Summarise key results with reference to study objectives	9
Limitations	19	Discuss limitations of the study, taking into account sources of potential bias or imprecision. Discuss both direction and magnitude of any potential bias	10
Interpretation	20	Give a cautious overall interpretation of results considering objectives, limitations, multiplicity of analyses, results from similar studies, and other relevant evidence	9-10
Generalisability	21	Discuss the generalisability (external validity) of the study results	9
**Other information**			
Funding	22	Give the source of funding and the role of the funders for the present study and, if applicable, for the original study on which the present article is based	3,6

Table S4: Cause of death as determined by InterVA and physician's review for 143 maternal deaths in the CLIP trials

	**Overall** (N=143)			**India** (N=16)			**Pakistan** (N=105)			**Mozambique** (N=22)		
	**PR**	**InterVA-4**	**InterVA-5**	**PR**	**InterVA-4**	**InterVA-5**	**PR**	**InterVA-4**	**InterVA-5**	**PR**	**InterVA-4**	**InterVA-5**
**Direct**	88 (61.5)	100 (69.9)	110 (76.9)	12 (75.0)	11 (68.8)	11 (68.8)	69 (73.3)	77 (73.3)	85 (81.0)	7 (32.0)	12 (54.5)	14 (63.6)
**Indirect**	39 (27.5)	37 (26.1)	28 (19.7)	4 (25.0)	4 (25.0)	3 (18.8)	23 (21.9)	25 (23.8)	19 (18.1)	12 (54.5)	8 (36.4)	6 (27.3)
**Specific CODs**												
1. Pregnancies with abortive outcome	1 (0.7)	0	1 (0.7)	1 (6.3)	0	0	0	0	1 (1.0)	0	0	0
2. Hypertensive disorders	23 (16.1)	25 (17.5)	24 (16.8)	2 (13.5)	4 (25.0)	2 (13.5)	19 (18.1)	19 (18.1)	18 (17.1)	2 (9.1)	2 (9.1)	4 (18.2)
3. Obstetric hemorrhage	38 (26.6)	69 (48.3)	82 (57.3)	5 (31.3)	6 (37.5)	9 (56.3)	29 (27.6)	53 (50.5)	63 (60.0)	4 (18.2)	10 (45.5)	10 (45.5)
4. Pregnancy-related infection	6 (4.2)	2 (1.4)	0	1 (6.3)	1 (6.3)	0	5 (4.8)	1 (1.0)	0	0	0	0
5. Other obstetric complications	16 (11.2)	4 (2.8)	3 (2.1)	3 (18.8)	0	0	12 (11.4)	4 (3.9)	3 (2.9)	1 (4.5)	0	0
Venous thromboembolism	8	0	0	1	0	0	6	0	0	1	0	0
Uterine inversion/rupture	2	0	1	0	0	0	2	0	1	0	0	0
Suicide	2	1	1	1	0	0	1	1	1	0	0	0
Obstructed labour	0	3	1	0	0	0	0	3	1	0	0	0
Other*	4	0	0	1	0	0	3	0	0	0	0	0
6. Unanticipated complications of management	4 (2.8)	0	0	0	0	0	4 (4.0)	0	0	0	0	0
7. Non-obstetric complications	39 (27.3)	37 (25.9)	28 (19.6)	4 (25.0)	4 (25.0)	3 (18.8)	23 (22.0)	25 (24.0)	19 (18.0)	12 (54.5)	8 (36.4)	6 (27.3)
Infectious disease	26	28	20	1	2	1	16	20	15	9	6	4
Respiratory	9	11	9	1	1	1	8	10	8	0	0	0
Gastrointestinal	3	1	0	0	0	0	2	1	0	1	0	0

	**Overall** (N=143)			**India** (N=16)			**Pakistan** (N=105)			**Mozambique** (N=22)		
	**PR**	**InterVA-4**	**InterVA-5**	**PR**	**InterVA-4**	**InterVA-5**	**PR**	**InterVA-4**	**InterVA-5**	**PR**	**InterVA-4**	**InterVA-5**
Malaria	5	4	1	0	1	0	1	1	0	4	2	1
HIV	2	8	7	0	0	0	0	4	4	2	4	3
Tuberculosis	2	3	1	0	0	0	2	3	1	0	0	0
Otherł	5	1	2	0	0	0	3	1	2	2	0	0
Cardiac disease	10	3	4	2	0	2	5	1	1	3	2	1
Liver disease	2	3	2	1	0	0	1	3	1	0	0	1
Other‡	1	3	2	0	2	0	1	1	2	0	0	0
8. Unknown/ undetermined	16 (11.2)	6 (4.2)	4 (2.8)	0	1 (6.3)	1 (6.3)	13 (12.4)	3 (2.9)	1 (1.0)	3 (13.6)	2 (9.1)	2 (9.1)
9. Coincidental causes	0	0	1 (0.7)	0	0	1 (6.3)	0	0	0	0	0	0
**COMCAT category**												
Traditions	-	-	1 (0.7)	0	-	-	-	-	1 (1.0)	-	-	-
Emergencies	-	-	68 (47.6)	2 (12.5)	-	13 (81.3)	-	-	45 (42.9)	-	-	10 (45.5)
Recognition	-	-	1 (0.7)	2 (12.5)	-	-	-	-	1 (1.0)	-	-	-
Resources	-	-	9 (6.3)	0	-	1 (6.3)	-	-	7 (6.8)	-	-	1 (4.5)
Health system	-	-	62 (43.4)	7 (43.8)	-	2 (12.5)	-	-	50 (35.0)	-	-	10 (45.5)
Inevitability	-	-		5 (31.3)	-	-	-	-	-	-	-	-
Multiple	-	-	2 (1.4)	0	-	-	-	-	1 (1.0)	-	-	1 (4.5)

CODs (causes of death), COMCAT (Circumstances Of Mortality CATegories), HIV (human immunodeficiency virus), InterVA (Interpreting VAs), NA (not applicable), PR (physician review), VA (verbal autopsy), VTE (venous thromboembolism)

** Other obstetric complications included amniotic fluid embolism (N=1), peripartum cardiomyopathy (N-=1), complications of intra-uterine fetal demise (N=1), dissiminated intravascular coagulation (N=1).*

Ł Other infections included meningitis (N=2), tetanus (N=1), hepatitis (N=1), measles (N=1), and infections not otherwise specified (N=3).

ǂ Other non-obstetric causes included stroke (N=1), breast neoplasm (N=1), asthma (N=2), and chronic obstructive pulmonary disease (N=2).

Table S5: Kappa statistics by country, for measurement of agreement between methods of assigned maternal COD for the 143 maternal deaths in the CLIP trials*

	**Overall**	**India**	**Pakistan**	**Mozambique**
**Direct/indirect/undetermined**				
Physician review vs InterVA-4	0.56 (0.43, 0.66)	0.56 (0.13, 0.98)	0.55 (0.39, 0.70)	0.52 (0.25, 0.80)
Physician review vs InterVA-5	0.44 (0.30, 0.57)	0.43 (0.03, 0.82)	0.41 (0.25, 0.57)	0.47 (0.21, 0.73)
InterVA-4 vs -5	0.72 (0.60, 0.84)	0.87 (0.64, 1.00)	0.67 (0.51, 0.83)	0.77 (0.54, 1.00)
**ICD-MM**				
Physician review vs InterVA-4	0.48 (0.38, 0.58)	0.47 (0.18, 0.77)	0.45 (0.34, 0.57)	0.63 (0.37, 0.89)
Physician review vs InterVA-5	0.36 (0.27, 0.46)	0.27 (0.04, 0.50)	0.35 (0.24, 0.45)	0.51 (0.25, 0.77)
InterVA-4 vs -5	0.69 (0.59, 0.79)	0.61 (0.32, 0.91)	0.68 (0.56, 0.80)	0.77 (0.55, 0.99)

CLIP (Community-Level interventions in Pre-eclampsia), COD (cause of death), ICD-MM (International Classification of Disease, Maternal Mortality), InterVA (Interpreting VAs), VA (verbal autopsy)

** A kappa statistic of <0.40 (red) was considered fair, 0.4-0.75 moderate (amber), and >0.75 good (green).*

Text S6: Data Sharing Statement

CLIP Trials Data Sharing Statement

The CLIP Trial data are de-identified participant-level data. Once the primary CLIP manuscripts, individual participant data meta-analysis, and papers based on the other pre-defined analyses are published as per the Statistical Analysis Plan (SAP), the data will be freely available to academically-active entities (e.g., universities, NGOs, multilaterals), with the CLIP Principal Investigator (Peter von Dadelszen) or named delegate as a named co-investigator, for the purposes of pregnancy-related research and within the limits of the informed consent obtained. Access will be through the CLIP Trials Data Access Committee*, contacted at 'PRE-EMPT@cw.bc.ca', as referenced on our website at 'https://PRE-EMPT.bcchr.ca'. A full data dictionary and all study documents will be available. Access will be through written application. When approved, a quote for the costs of preparing the data will be provided to the applicant.

By submitting an application form, the investigator agrees that s/he has read, understood and agrees to the terms and conditions below:

1. S/he is an academically-active researcher affiliated with an entity able to engage in a data transfer agreement;
2. S/he warrants that the information entered is to the best of her/his knowledge full and correct;
3. S/he agrees that the Data Sharing Agreement will only be used for the specific project outlined in the application;
4. S/he represents that s/he has obtained the necessary approvals to transfer the data and/or receive the data under this Data Sharing Agreement;
5. S/he understands that the responses provided will form part of a legally-binding document;
6. S/he understands that the Agreement is not valid until a fully-executed copy, with signatures from all parties, is emailed to PRE-EMPT@cw.bc.ca); and
7. S/he understands that no modifications can be made to the Data Sharing Agreement and if modifications are made, the Data Sharing Agreement will be rendered invalid.

There is no pregnancy-specific repository for us to access, but once the primary papers for the CLIP Trials have been published, we will be depositing a copy of our data in the HBGDki repository at the Bill & Melinda Gates Foundation, our funder. The p<u>ermitted uses and disclosures of these data are as follows:</u>

1. The Foundation will limit the use and disclosure of the CLIP data to conduct research related to achieving the goals of the Foundation as represented above. The Foundation may also de-identify the data set and aggregate it with other de-identified information.
2. The Foundation will restrict access to the CLIP data to individuals involved in the Foundation's research who have a need to access the CLIP data to carry out their duties as they relate to the Permitted Uses and Disclosures identified above, and any such access will be consistent with the assurances and obligations set forth in this Agreement. The Foundation will use appropriate safeguards to prevent use or disclosure of the CLIP data other than as permitted by this Agreement.
3. The Foundation will report to HBGDki Collaborator any use or disclosure of the CLIP data not provided for by this Agreement of which the Foundation becomes aware.

4. The Foundation will ensure that any agents, including subcontractors, to whom it provides the CLIP data, if any, agree to the same restrictions and conditions that apply to the Foundation with respect to such information.

** The **Data Access Committee** is made up of the following individuals: Peter von Dadelszen and Laura A. Magee (King's College London, UK); Zulfiqar A Bhutta (Aga Khan University, Karachi, Pakistan and the Hospital for Sick Children, Toronto, Canada); Rahat N Qureshi (Aga Khan University, Karachi, Pakistan); Ashalata A Mallapur (S Nijalingappa Medical College, Bagalkote, India); Mrutyunjaya B Bellad and Shivaprasad Goudar (KLE Academy of Higher Education and Research's JN Medical College, Belagavi, India); Khátia Munguambe, Charfudin Sacoor, and Esperança Sevene (Centro de Investigação em Saúde da Manhiça, Manhiça, Mozambique)*
